# Social and contextual factors that influence HIV risk behaviors among indigenous MSM in the Peruvian Amazon

**DOI:** 10.1186/1742-4690-9-S1-P112

**Published:** 2012-05-25

**Authors:** Isaac E Alva, E Roberto Orellana

**Affiliations:** 1 Universidad Peruana Cayetano Heredia, Lima, Peru

## Introduction

Men who have sex with men (MSM) face a significantly higher risk of HIV infection than the general population around the globe. In Peru, HIV prevalence among MSM range from 14% to 23%, with Lima, the capital, and port cities in the Amazonian region being the most affected. Recent studies found that indigenous MSM who leave their villages for cities along the Amazon River and its tributaries, engage in high risk behaviors such as high alcohol consumption and unprotected sex with mestizo (non- indigenous) MSM. This study examined social and contextual factors associated with risky behaviors among indigenous MSM in the Peruvian Amazon.

## Materials and methods

During a 5-month period in 2009-2010, we purposively recruited indigenous MSM. The study took place in several port cities throughout the Amazon region. Semi-structured in-depth interviews were conducted with indigenous men, who consented to voluntarily participate in the study.

## Results

We interviewed 34 MSM with an average age of 26 years. They represented 8 different ethnic groups. In most situations, when family and community members learned about the participants' sexuality, discrimination and violence ensued. Participants reported being beaten up by their relatives. Sometimes, community councils were held to decide on their fate. Council decisions ranged from forcing the person to hard (manly) labor, to undertake traditional medicine treatments, to expulsion. Participants saw their migration to the city as an escape from oppressive forces in the community. In the city, many reported being in abusive relationships with other men. Lacking appropriate education and technical skills, many participants engaged in sex work as a way of making a living.

## Conclusions

A great deal of discrimination, isolation and lack of social support was experienced by most participants. Besides individual-level interventions, HIV prevention programs should take these factors into account and design programs that increase social support, enhance community building and reduce stigma.

